# Diamond Needles Actuating Triple-Walled Carbon Nanotube to Rotate via Thermal Vibration-Induced Collision

**DOI:** 10.3390/ijms20051140

**Published:** 2019-03-06

**Authors:** Hui Li, Aiqin Wang, Jiao Shi, Yongjian Liu, Gao Cheng

**Affiliations:** 1 School of Highway, Chang’an University, Xi’an 710064, China; lihui2210@yeah.net (H.L.); chengg@chd.edu.cn (G.C.); 2 College of Water Resources and Architecturally Engineering, Northwest A&F University, Yangling 712100, China; waq18700944239@126.com

**Keywords:** nanomotor, carbon nanotube, diamond, molecular dynamics

## Abstract

A rotary nanomotor is an essential component of a nanomachine. In the present study, a rotary nanomotor from wedged diamonds and triple-walled nanotubes was proposed with the consideration of boundary effect. The outer tubes and mid-tubes were used as nanobearing to constrain the inner tube. Several wedges of the diamond were placed near the inner tube for driving the inner tube to rotate. At a temperature lower than 300 K, the inner tube as the rotor had a stable rotational frequency (SRF). It is shown that both the rotational direction and the value of SRF of the rotor depended on the temperature and thickness of the diamond wedges. The dependence was investigated via theoretical analysis of the molecular dynamics simulation results. For example, when each diamond wedge had one pair of tip atoms (unsaturated), the rotational direction of the rotor at 100 K was opposite to that at 300 K. At 500 K, the rotating rotor may stop suddenly due to breakage of the diamond needles. Some conclusions are drawn for potential application of such a nanomotor in a nanomachine.

## 1. Introduction

Since discovered in 1991 [1], carbon nanotubes (CNTs) have attracted much attention [2,3,4]. After about three decades of research, the excellent physical properties of CNTs have been revealed gradually. Owing to two excellent mechanical features, carbon nanotubes (CNT) are popular in the design of dynamic nanodevices, e.g., oscillators [2,3,5,6,7], resonators [8,9,10], bearings [11,12,13], nano strain sensors [14,15], and nanomotors [16,17,18,19,20,21,22]. The two mechanical properties are superhigh in-shell strength [23] and inter-shell superlubrication [11,24,25] due to the special electron configuration of carbon atoms in CNT. For example, the covalent bonds formed between s-electrons of atoms lead to super high in-shell strength which guarantees stability of the CNT component working in a nanodevice. The delocalized anti-bond p-electron of each carbon atom causes inter-shell superlubrication which leads to extremely low energy dissipation during the motion of CNT components [26].

For nanomotors from CNTs, especially the rotary nanomotors, the method to drive the rotor to rotate presents challenges. So far, three major types of driving methods have been proposed. The first one is due to temperature change or its nonuniform distribution. For example, in the work by Barreiro et al. [19], the long inner tube has a temperature difference between two edges, and a temperature gradient was generated to actuate the motion of the short outer tube attached to cargo. In 2014, Cai et al. [22] discovered that even without a temperature gradient, the inner tube can still obtain a gigahertz rotation in an outer tube. In 2016, they [27] developed an accurate method to control the rotational direction and frequency of the rotor by introducing inwardly radial deviation of some atoms at the edges of the stator. The second method is to place the CNT-motor in an external field such as electronic field. For example, Tu and Hu [18] proposed a theoretical model of a rotary nanomotor from few-nanometered CNTs. In their model, the motion of the short outer tube on a long inner tube was actuated by an external electronic field. Wang et al. [20] built a complicated rotor from CNTs and fullerenes; through periodic recharging and discharging of the carbon arms in the rotor, the rotor was driven to rotate in an external electronic field. In the final method, the rotor of a nanomotor is driven to rotate by nanofluid. For instance, Kang and Hwang [17] built a rotary nanodevice by mimicking of the model of hydroturbine. The blades attached to the bearing can be driven to move by the nanofluid in a capsule. Similarly, Prokop et al. [21] suggested using some type of gas to actuate the rotation of a nanomotor.

**Figure 1:**
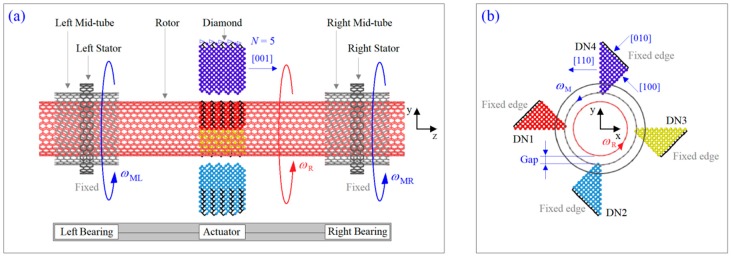
Schematic model of a diamond-excited nanomotor from tri-walled carbon nanotubes. (**a**) Side-view of system, (**b**) axial-view of system.

Comparing the above models of nanomotors, the double-walled CNTs based nanomotor in refs. [18,19,27] can be easily fabricated. Without the limitation on the chirality of CNTs, the nanomotor model proposed by Tu and Hu [18] is the simplest one among the models. The model presented by Cai et al. [27] is easily fabricated and the temperature field with or without gradient can be used to drive the rotation of the rotor. The drawback could lie in the accurate control of the inwardly radial deviation of the edge atoms. Further study [25] also indicates that the output rotation of the rotor depends on the radial deviation of atoms. Besides, the boundary effect cannot be ignored. For example, the rotor should be avoided to contact the fixed atoms directly. Therefore, there is space to develop new methods to build a rotary nanomotor. In this study, we choose triple-walled CNTs as rotary components in the nanomotor (Figure 1). The short outer tubes are fixed as stators. The two mid-tubes together with the two stators pose as nanobearings, which will constrain the motion of the inner tube (i.e., the rotor). The nanotubes in bearing are not the actuator of the inner tube. Moreover, constrained outer tubes have a slight boundary effect on the rotation of the inner tube. For a nanomotor, it needs actuator(s). Therefore, we employ a triple-walled CNT system in this work. Considering the super high hardness of diamond, near the rotor we used four diamond wedges as actuators. In Section 2, some molecular dynamics simulation results are provided to reveal the dynamic response of the rotor and the two mid-tubes. Details are given in Section 3. Finally, we draw some conclusions for potential application of the present method in the design of a nanomotor.

## 2. Results and Discussion

### 2.1. Effects of Temperature and Thickness of Diamond on the Rotor’s Rotation

Using the approach of molecular dynamics simulation, we obtain the histories of *ω*_R_ of the rotor constrained by thin stators at different conditions. According to the historical curves of *ω*_R_ shown in 0, we know that the rotor has a stable rotational frequency (SRF) after no more than 4 ns at 100 K (0a). By comparing the curves at 100 K, we conclude that the value of SRF is obviously higher when the rotor is driven by thicker diamond wedges. However, the values of SRF have slight differences when *N* > 3. For example, the rotor’s SRFs are −49.64 GHz, −50.25 GHz, and −50.85 GHz driven by the diamond wedges with *N* = 4, 5, and 6, respectively. The negative value of SRF means the rotor rotates in clockwise direction. If the SRF is positive, the rotor has a counter-clockwise rotation. But clearly, at 100 K the rotor’s rotational direction is independent of the thickness of the diamond wedges.

**Figure 2:**
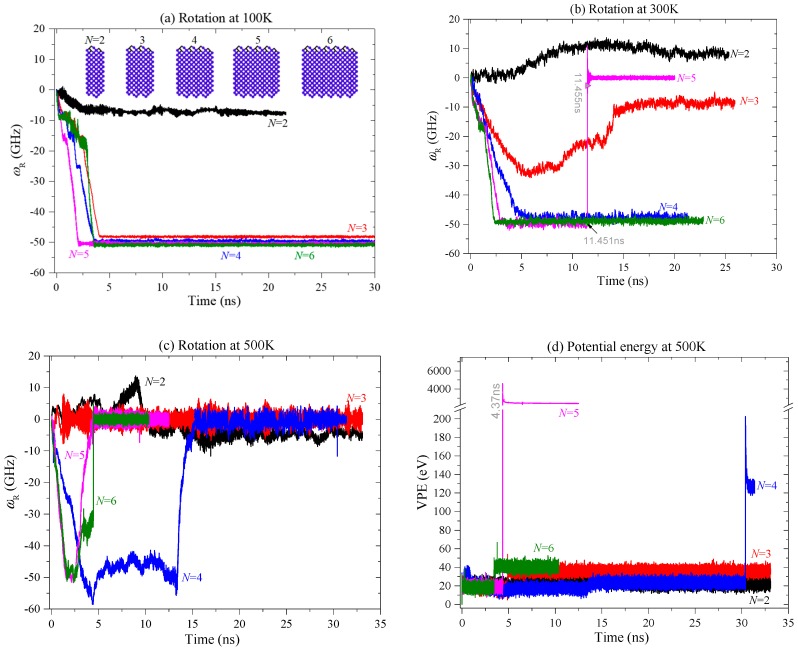
Histories of rotor rotation at different conditions. (**a**) The rotational frequency of rotor driven by different DNs at 100 K, (**b**) at 300 K, and (**c**) at 500 K. (**d**) Variation of potential energy (VPE) of the system at 500 K.

At room temperature, the rotor rotation (Figure 2b) is different from that at 100 K. The first difference is that the rotor at 300 K needs more time to exhibit a stable rotation than at 100 K. Secondly, *ω*_R_ has higher amplitude of fluctuation in the simulation process, especially when the rotor is driven by the diamond wedges with *N* = 3, as it has a U-shaped curve in the first 14 ns. When the edges of the mid-tubes get closer to the edges of the stators, it is noted that the frictional resistance from the stators leads to deceleration of the mid-tubes. Further, the mid-tubes prevent acceleration of the rotor via inter-shell friction. This is the reason for the U-shaped variation process of *ω*_R_. It also demonstrates that the stability of the diamond wedges is lower at higher temperatures.

The third difference is that the rotor is in an anti-clockwise rotation when *N* = 2. The reason is that, as each diamond wedge only has one pair of tip atoms (unsaturated), they are subject to weaker compression from the rotor at lower temperatures. Meanwhile, the rotor induces lower impact force on the tip atoms on wedges during collision between the wedges and the rotor at lower temperatures. Such lower interaction cannot induce a large rotational angle of the covalent bond between each pair of tip atoms. However, at higher temperatures, the rotor has higher thermal expansion, which leads to stronger compression on the tips atoms, and the impact force from the rotor is also higher. Hence, the bonds at wedge tips have a large rotational angle. It is due to the rotation of the bonds; the wedge tips provide reverse repulsion onto the rotor at 300 K. Therefore, the rotor rotates in a clockwise direction at 100 K, but in an anti-clockwise direction at 300 K.

**Figure 3:**
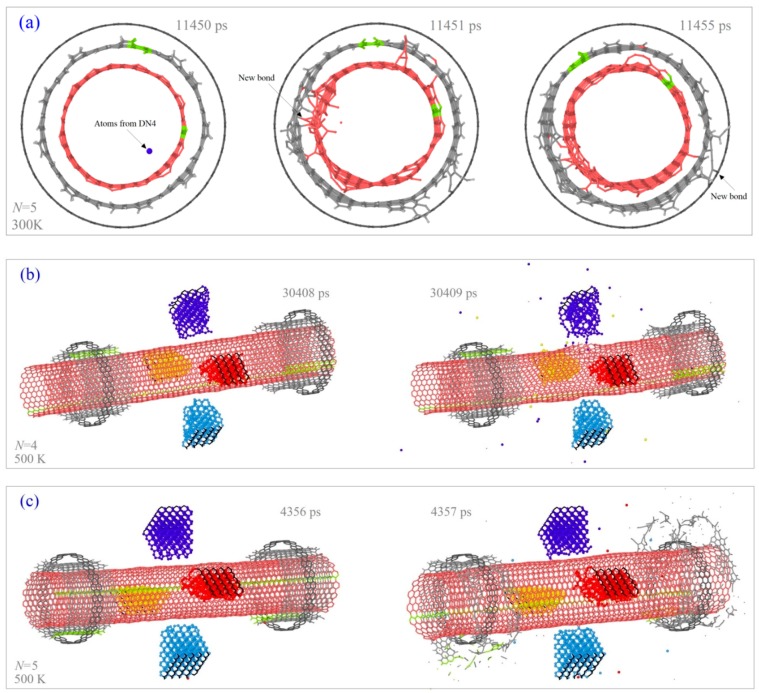
Snapshots of the system under different conditions. (**a**) Bonding process among the components in system with *N* = 5 at 300 K. (**b**) Sudden collapse of DNs with *N* = 4 at 500 K. (**c**) Sudden collapse of mid tubes in the system with *N* = 5 at 500 K.

**Figure 4:**
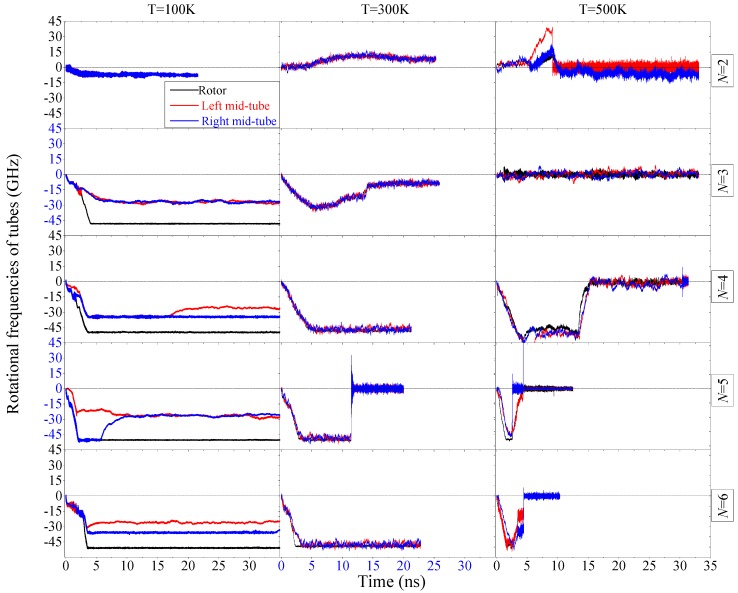
Historical curves of rotational frequencies of the rotor and the related mid-tubes under different conditions. The curves in the left column are obtained at 100 K, those in the middle column are obtained at 300 K, and the data in the right column are obtained at 500 K. The curves in the top row are of the rotor driven by the DNs with *N* = 2. The bottom row contains the results with respect to *N* = 6.

The fourth difference is that the rotor has sudden stoppage at 300 K when it is actuated by the diamond wedges with *N* = 5 (the pink curve in Figure 2b). Comparing the snapshots of the system at 11,450 ps, 11,451 ps (Figure 3a), we find that the left edges of the rotor, the mid-tube, and the left stator have obvious differences within 1ps. For example, at 11,450 ps the topology of all the edges is not changed, i.e., the edges have no bond being broken or generated from their initial configurations. However, at the next 1ps, the mid-tube is bonded with both the rotor and the stator. Due to the super high strength of the new C-C bonds, the rotor’s rotational frequency drops rapidly. For instance, after only 4 ps (i.e., at 11455 ps), the value of *ω*_R_ jumps from ~ −50 GHz to ~ 2 GHz. And the rotor finally stops rotating. It demonstrates that sudden stoppage of the high-speed rotating rotor is mainly caused by the injection of one or more atoms which escape from the broken diamond wedges. Hence, at room temperature, the diamond wedges’ stability is lower than that at 100 K. Without stable diamond wedges, the stability of the rotor rotation cannot be guaranteed. Comparing the three models with respect to *N* = 4, 5 and 6 (Figure 2b), we find that only the rotor driven by the DN with *N* = 5 stops suddenly. The reason is that thermal vibration at the tip of the diamond needle causes the atoms to have higher potential energy than the internal atoms. The C-C bonds among the tip atoms are easily broken. At a finite temperature, thermal vibration of atoms combining their collision with the atoms on the rotor may lead to breakage of the tip bonds. In controlling the temperature of the system, the velocities of atoms are modified using a Nose-Hoover thermostat. Hence, random effect is one of reason for both breakage of the tip bonds and sudden stoppage of the rotor. But the tendency is that at higher temperatures, random effect is stronger, the bond breakage happens easier, and the rotor stops rotating earlier (Figure 4).

The final difference is that the rotor at 300 K rotates slower than at 100 K. For example, the value of SRF of the rotor with respect to *N* = 4 is ~−47.67 GHz at 300 K, while ~−49.64 GHz at 100 K. When *N* = 6, the value of SRF is ~−48.9 GHz at 300 K, while ~−50.85 GHz at 100 K. The reason is that the stators provide higher friction to the mid-tubes at a higher temperature.

To verify the conclusion that the stability of the diamond wedges becomes lower at higher temperatures and influences the rotor rotation, we also test the nanomotor at 500 K. According to the curves of *ω*_R_ in Figure 2c, one can find that the rotor has no rotation except when *N* = 2. The reason is that a thick broken diamond wedge has more atoms escaping, and the atoms have a higher chance to damage the high-speed rotating tubes. Further, the damaged tubes will covalently bond with the stator. From the VPE curves of the system at different conditions in Figure 2d, we know that the system with respect to *N* = 5 has the worst damage according to the highest jumping of VPE near 4.37 ns. This can be verified from their seriously damaged configurations shown in Figure 3b,c.

### 2.2. Rotation of Mid-Tubes in Bearings

In the model of the nanomotor mentioned above, the two mid-tubes are constrained only on its translational motion along the x-/y-direction; hence, rotation of the two tubes can happen. Figure 4 shows the rotation of the two tubes together with the rotor. From the interaction among the tubes, the rotor is driven to rotate by the diamond wedges. The mid-tubes are driven by the rotor, but resisted by the stators due to the existence of inter-tube friction. Hence, the rotation of the two mid-tubes should be no faster than that of the rotor. According to the statistical results in Table 1, the rule is not violated in most cases. Exception happening, e.g., at *N* = 2 with T = 100 K or *N* = 3 with T = 300 K, is mainly caused by the length of the statistical duration. Besides, the ratios of *ω*_M_ to *ω*_R_ are just slightly different from 1.0. Hence, in the two cases, the mid-tubes are not in over-speeding rotation [28].

At 100 K, if *N* > 2, the rotational speed of the two mid-tubes is just about half of that of the rotor. Even in some cases, e.g., *N* = 4 or 6, the two mid-tube have different rotational speeds. The reason is that the rotor oscillates along the z-direction during rotation. When an edge of the rotor moves closer to the outer edge of a mid-tube, by considering the effect of edge potential barrier [29,30], the rotor can provide stronger friction for the mid-tube which will be driven to rotate faster than the other mid-tube. This phenomenon applies also in asynchronous rotation transmission system [31].

At 300 K or 500 K, the three tubes may stop rotating after a few nanoseconds. The reason for the sudden stoppage of the rotor has been given in the above discussion. The sudden stoppage of the two mid-tubes causes resistance from both the rotor and stator even if the mid-tube is not covalently bonded with one of them. Hence, we conclude that if the rotor stops suddenly, the two mid-tubes stop rotating synchronously.

### 2.3. Rotation of the Rotor in Wider Stators

**Figure 5:**
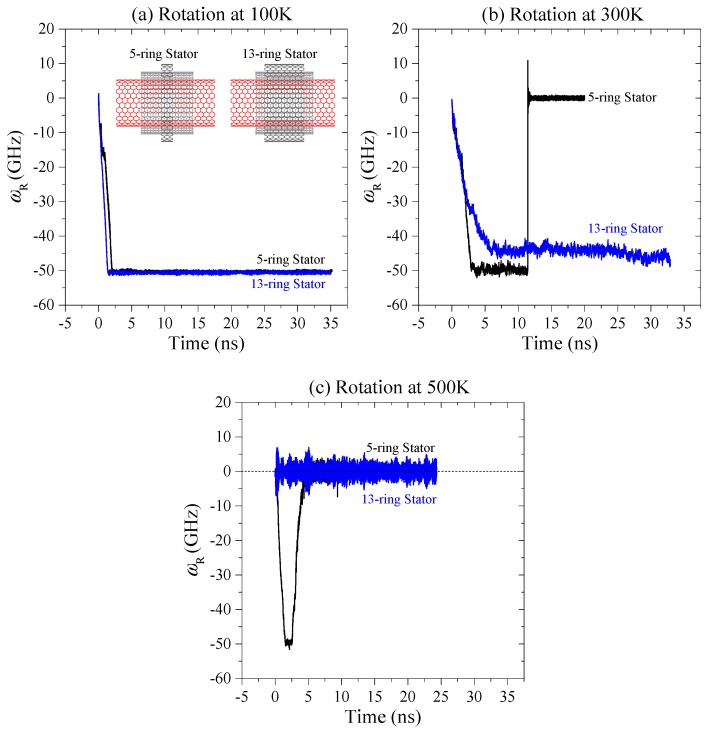
Historical curves of *ω*_R_ of the rotor driven by the diamond wedge with *N* = 5 at different temperatures. (**a**) Rotational frequency of rotor at 100 K, (**b**) at 300 K, and (**c**) at 500 K.

In the analysis above, we found that the rotation of the rotor may stop suddenly. With the exception of the instability of the diamond wedges, using the thin stators to constrain the mid-tubes may be another reason. Briefly, constrained by the narrow stators (5-ring stators in Table 2), the two mid-tubes may have drastically eccentric rotation. At an eccentric rotation, the edges of the two mid-tubes have a high chance of getting close to the edges of the rotor and the stators. It means that covalent C-C bonds can be easily generated between the mid-tubes and the remaining two tubes. Hence, we use wide stator (13 rings in Table 2) to constrain the mid-tubes. The related histories curves of rotation of the rotor are drawn in Figure 5.

At 100 K, the rotor has stable rotation after ~2 ns for both cases (Figure 5a), and the value of SRF of the rotor is slightly dependent on the width of the stators. It is because the internal saturated atoms in the stators provide slight friction on the mid-tubes [25,29]. At 300 K (Figure 5b) that the effect of the width of the stators on rotation of the rotor is obvious. For example, the rotor rotation stops suddenly after 11.45 ns when the rotor is constrained by the narrow stators. However, the rotor rotates in a stable state. Unfortunately, at 500 K (Figure 5c), the wide stators still cannot maintain the rotor in a stable rotation. A conclusion can be drawn that the thick stators can improve the stability of rotor rotation at room temperature or lower.

## 3. Model and Methodology

### 3.1. Model of a Rotary Nanomotor

Consider a nanomotor model as shown in Figure 1. The system consists of three major components, i.e., the inner tube as the rotor, the two identical nanobearings, and the actuator consisting of four diamond wedges. The outer tubes in the two nanobearings are fixed as stators. The mid-tubes protect the rotor from direct action of the stators. The two edges of each mid-tube are hydrogenated. The actuator provides driving force for the rotor during their collision at a finite temperature. Each diamond wedge has *N* layers along the [001] lattice direction, e.g., *N* = 5 in Figure 1a. The [001] lattice direction is parallel to the z-axis. The outer tubes with different widths are adopted in the system. The detailed parameters are listed in Table 2.

### 3.2. Methodology

To illustrate the dynamic response of the rotor and the two mid-tubes, the molecular dynamics approach is adopted, and the simulations are carried out via the open source code LAMMPS [32,33]. Each simulation contains six major steps as follows. Firstly, create a model with specified geometrical parameters, then put the model in a simulation box with solid boundary conditions along the three dimensions. In this process, one can form the CNTs first, and then move the prepared diamond wedges toward the rotor simultaneously. Secondly, adjust the shapes of the tubes by minimizing the potential energy of the system. After adjustment, both the edge effect and inter-shell interaction can be smoothed, and a reasonable initial configuration of the system can be obtained. Thirdly, specify the atoms in the system with the initial velocities which satisfy the physical state of the particle system. Fourthly, fix the atoms in the two stators or at the edges of the diamond wedges (Figure 1). Fifthly, put the system in a NVT ensemble with given temperature. Where N, V, and T are the number of unfixed particles, the volume and the temperature of the system, respectively. They keep unchanged in simulation. Finally, calculate the rotational frequencies of the rotor and the two mid-tubes, and record the transient configurations of the system for post-processing.

In the simulation above, the integral time step is set at 0.001 ps. The temperature is controlled by a Nose-Hoover thermostat [34]. In this study, 100 K as low temperature, 300 K as room temperature, and 500 K as high temperature are involved in simulations. The interaction among atoms in the system is described by AIREBO potential [35]. It is known that a stable system must have a minimum potential energy. To describe the stability of a nanosystem, we calculate the variation of potential energy (VPE) during simulation. The value of VPE can be obtained by subtracting the initial potential energy from the current value. Briefly, both bond breakage and departure of the atoms from the system lead to VPE increase of the system.

### 3.3. Mechanism of Rotation

At a finite temperature, the unfixed atoms in the model, e.g., Figure 1, have thermal vibration, and the amplitude of vibration is greater at a higher temperature. When the distance between an atom on the rotor and the tip atoms on a diamond wedge is less than 0.34 nm, the atom is subjected to a repulsive force from the tip atoms. The force is described by the Lennard-Jones potential [36]. In this study, the initial value of gap (Figure 1b) is set to 0.3 nm (< 0.34 nm). Hence, the rotor always has one or more atoms colliding with the tip atoms on diamond wedges. From the layout of the diamond wedges shown in Figure 1b, we know that the atoms on the (110) lattice plane almost generate a repulsive force along the radial direction. Hence, the tangent component of the repulsive force is negligible. However, the repulsive force from the atoms on the (100) lattice plane has a non-zero component along the tangent direction of the rotor. The tangent component drives the rotor to rotate. These are the mechanics for the present model of a rotary nanomotor.

During rotating, the rotor is subjected to the inter-shell friction from both the stators and the mid-tubes [31]. Moreover, the friction increases with their relative speeds [26]. When the friction-induced moment can balance the active moment from the actuators, i.e., the diamond wedges, the rotor is in a stable rotation. The stable value of *ω*_R_ can be expressed as

(1) $$\omega_{R}\left(t\to \infty \right)=\int_{0}^{t}\frac{\left[M_{\mathrm{DN}}\left(s\right)-M_{\mathrm{SR}}\left(s\right)-M_{\mathrm{MR}}\left(s\right)\right]}{J_{Z}}ds$$

where, *M*_DN_ is the active moment, *M*_SR_ and *M*_MR_ are the resistant moment from the stators and the mid-tube, respectively. *J*_z_ is the mass inertia of rotation of the rotor about the z-axis. Actually, the stable rotation can be reached after only few nanoseconds. Similarly, the rotational frequencies of the two mid-tubes can also be given as,

(2) $$\omega_{\mathrm{ML}}\left(t\right)=\int_{0}^{t}\frac{\left[M_{\mathrm{RML}}\left(s\right)-M_{\mathrm{SML}}\left(s\right)\right]}{J_{Z}}ds$$

where *M*_RML_ is the active moment from the rotor and *M*_SML_ is the resistant moment from the left stator.

## 4. Conclusions

In this study, we proposed a model of rotary nanomotor from diamond and tri-walled nanotubes. The outer tubes are fixed as stators. The diamond needles were placed 0.3 nm away from the inner tube, which acted as a rotor. At a thermostat with specified temperature, the dynamic response of the rotor and the mid-tubes depended on both the temperature and the thickness (*N*) of the diamond wedge. According to the numerical simulation results, some conclusions are drawn as follows:

(1) At 100 K, the rotor had a more stable rotational frequency (SRF) when it was driven by thicker diamond needles. When *N* > 3, the values of SRF are slightly different.
(2) When the rotor is driven by the diamond needles with *N* = 2, its rotational direction at 100 K is opposite that of 300 K. The reason is that the C-C bonds at the tips of the wedged diamond had a high rotational angle at 300 K. The deformed diamond tips provide reverse repulsion for the rotor to rotate.
(3) At 300 K or a higher temperature, the rotating rotor may stop suddenly. In particular, at 500 K, the rotor cannot reach a stable rotation before stopping. Interrupt of the high-speed stable rotation of the rotor was mainly caused by the intrusion of one or more atoms which escaped from broken diamond needles.
(4) At 100 K, the two mid-tubes may have different rotational frequency from that of the rotor. It is caused by the edges of tubes having stronger interaction when they stayed close to each other. At 300 K, the two mid-tubes rotated approximately synchronously with the rotor. If the rotor stopped suddenly, the two mid-tubes stopped simultaneously.
(5) Using thicker stators to constrain the mid-tubes at room temperature, the stability of rotor rotation can be improved.

These conclusions can be taken as guidelines for fabrication of such rotary nanomotors. For example, to have a stable diamond needle, the environment temperature should be controlled to be no obviously higher than room temperature. If we need a rotary nanomotor working at a higher temperature, a stable actuator should be designed. In our future work, we will focus on finding out more about stable materials to replace the diamond actuator.

## Figures and Tables

**Table 1:** Stable rotational frequency (SRF) of the rotor and the mid tubes at different conditions. The statistics is fulfilled in the last 5 ns of each case.

SRF	100 K					300 K				
	*N* = 2	*N* = 3	*N* = 4	*N* = 5	*N* = 6	*N* = 2	*N* = 3	*N* = 4	*N* = 5	*N* = 6
*ω*_R_/GHz	−7.18	−48.19	−49.64	−50.25	−50.85	8.16	−8.57	−47.67	0	−48.90
*ω*_ML_/GHz	−7.18	−26.80	−25.73	−26.64	−25.94	7.85	−8.69	−46.38	0	−47.26
*ω*_MR_/GHz	−7.19	−26.68	−34.46	−26.29	−35.71	8.01	−8.48	−46.82	0	−47.66
ratio1	1.000	0.556	0.518	0.530	0.510	0.962	1.014	0.973	1.00	0.966
ratio2	1.001	0.554	0.694	0.523	0.702	0.982	0.989	0.982	1.00	0.975

Note: ratio1 = *ω*_ML_ /*ω*_R_, ratio2 = *ω*_MR_ /*ω*_R_.

**Table 2:** Parameters of nanomotor models involved in simulations.

Component	(n, m)	Radius/nm	Length/nm	Ring/Layer Number	Number of Atoms	z-Distance/nm
Rotor	(15, 15)	1.017	13.7733	113	3390	/
Mid-tube	(20, 20)	1.356	2.2136	19	1420C + 80H	Between both mid-tubes: 8.0
Stator	(25, 25)	1.695	0.4919/1.4759	5/13	250/650	Between both stators: 9.7216/8.7376
Diamond	[001]//z	[1−10]//r	[100] edge = 1.358	*N* = 2/3/4/5/6	680/1036/1392/1748/2104	Between a tip and the rotor: gap = 0.3

Note: “//z” and “//r” represent parallel to the z-/r-directions, respectively.
